# Intranasal administration of ceramide liposome suppresses allergic rhinitis by targeting CD300f in murine models

**DOI:** 10.1038/s41598-024-58923-w

**Published:** 2024-04-10

**Authors:** Takuma Ide, Kumi Izawa, Wahyu Diono, Anna Kamei, Tomoaki Ando, Ayako Kaitani, Akie Maehara, Akihisa Yoshikawa, Risa Yamamoto, Shino Uchida, Hexing Wang, Mayuki Kojima, Keiko Maeda, Nobuhiro Nakano, Masahiro Nakamura, Toshiaki Shimizu, Hideoki Ogawa, Ko Okumura, Fumihiko Matsumoto, Katsuhisa Ikeda, Motonobu Goto, Jiro Kitaura

**Affiliations:** 1 Atopy (Allergy) Research Center, Juntendo University Graduate School of Medicine, 2-1-1 Hongo, Bunkyo-Ku, Tokyo, 113-8421 Japan; 2 Department of Otorhinolaryngology, Juntendo University Graduate School of Medicine, 2-1-1 Hongo, Bunkyo-Ku, Tokyo, 113-8421 Japan; 3 Department of Materials Process Engineering, Nagoya University, Furo-Cho, Chikusa-Ku, Nagoya, 464-8603 Japan; 4 Department of Science of Allergy and Inflammation, Juntendo University Graduate School of Medicine, 2-1-1 Hongo, Bunkyo-Ku, Tokyo, 113-8421 Japan; 5 Department of Gastroenterology Immunology, Juntendo University Graduate School of Medicine, 2-1-1 Hongo, Bunkyo-Ku, Tokyo, 113-8421 Japan; 6 Department of Pediatrics and Adolescent Medicine, Juntendo University Graduate School of Medicine, 2-1-1 Hongo, Bunkyo-Ku, Tokyo, 113-8421 Japan; 7 Department of Immunological Diagnosis, Juntendo University Graduate School of Medicine, 2-1-1 Hongo, Bunkyo-Ku, Tokyo, 113-8421 Japan

**Keywords:** Molecular medicine, Acute inflammation, Experimental models of disease

## Abstract

Allergic rhinitis (AR) is caused by type I hypersensitivity reaction in the nasal tissues. The interaction between CD300f and its ligand ceramide suppresses immunoglobulin E (IgE)-mediated mast cell activation. However, whether CD300f inhibits the development of allergic rhinitis (AR) remains elusive. We aimed to investigate the roles of CD300f in the development of AR and the effectiveness of intranasal administration of ceramide liposomes on AR in murine models. We used ragweed pollen-induced AR models in mice. Notably, CD300f deficiency did not significantly influence the ragweed-specific IgE production, but increased the frequency of mast cell-dependent sneezing as well as the numbers of degranulated mast cells and eosinophils in the nasal tissues in our models. Similar results were also obtained for MCPT5-exprssing mast cell-specific loss of CD300f. Importantly, intranasal administration of ceramide liposomes reduced the frequency of sneezing as well as the numbers of degranulated mast cells and eosinophils in the nasal tissues in AR models. Thus, CD300f–ceramide interaction, predominantly in mast cells, alleviates the symptoms and progression of AR. Therefore, intranasal administration of ceramide liposomes may be a promising therapeutic approach against AR by targeting CD300f.

## Introduction

Allergic rhinitis (AR) is caused by type I hypersensitivity reaction in the nasal tissues. AR is traditionally classified as seasonal and perennial, depending on the kind of allergen. Pollens such as ragweed (RW) pollen and house dust mites are major allergens of seasonal and perennial AR, respectively. Symptoms of AR include sneezing, watery rhinorrhea, and nasal congestion. The allergens invade the nasal mucosa and causes allergic sensitization, resulting in allergen-specific immunoglobulin E (IgE) production. Crosslinking of allergen-specific IgE by the same allergens triggers the degranulation of nasal mast cells, leading to the release of chemical mediators such as histamine. This early-phase response induces sneezing and watery rhinorrhea. Sneezing is caused because of the histamine-mediated irritation of sensory nerves in the nasal tissues. The subsequent late-phase response, characterized by infiltration of inflammatory cells like eosinophils into the nasal tissues, induces nasal congestion with epithelial damages. T helper type 2 (Th2) cells play a vital role in this late or chronic phase response of AR^1–4^.

Intranasal corticosteroid and/or histamine H_1_ receptor antagonists are commonly used in the treatment of AR. However, the symptoms of AR are difficult to control, which negatively affects the quality of life. Moreover, since the prevalence of AR has been increasing worldwide, there is an urgent need to identify potential therapeutic targets and develop more effective and safer therapeutic strategies against AR^1,2^.

The CD300 glycoproteins are a family of cell surface molecules that orchestrate diverse cellular processes through their paired activating and inhibitory receptor functions. They are also called CMRF-35-like molecules (CLM), leukocyte mono-immunoglobulin-like receptor (LMIR), or myeloid mono Ig-like receptor (MAIR)^5–15^. CD300f/CLM-1/LMIR3 is an immune receptor primarily expressed in myeloid cells. CD300f contains an immunoglobulin-like domain in its extracellular region and immunoreceptor tyrosine-based inhibitory and switch motif (ITIM and ITSM) in its cytoplasmic region^6,8,9^. Structural lipids such as ceramides and phosphatidylserine have been recently identified as ligands for CD300f^9,16–20^. The interaction between CD300f and extracellular ceramides inhibits high-affinity IgE receptor (FcεRI)-mediated mast cell activation and anaphylactic responses in mice via phosphorylation of ITIM and ITSM, which in turn can recruit SH2-containing protein tyrosine phosphatase 1 (SHP-1) and SHP-2^9,16,17,21,22^. In addition, the CD300f–ceramide interaction suppresses the Toll-like receptor 4 (TLR4)-, P2X7-, or Mrgprb2-mediated mast cell activation^23–27^. The intravenous or subcutaneous administration of ceramide liposomes, that have been prepared by using an extruder at the time of use, increases the levels of CD300f ligands in vivo, and suppresses the mast cell-dependent inflammatory responses^9,16,17,21–27^. Further, the binding of CD300f to phosphatidylserine exposed on apoptotic/dead cells is involved in their clearance by phagocytes^18,19^, while it negatively regulates eotaxin-induced eosinophil responses^20^. Additionally, CD300f positively regulates an activating signal under certain circumstances^28,29^. However, the roles of CD300f in the development of AR remain unknown.

In the present study, we used murine models^3,4,30,31^ of AR induced by RW pollen in wild-type (WT) and *CD300f*^*−/−*^ mice and demonstrated that CD300f suppresses the symptoms of AR as well as mast cell degranulation and eosinophil accumulation in the nasal tissues. Importantly, the intranasal administration of ceramide liposomes improves the symptoms of AR by targeting CD300f, which may provide a novel therapeutic strategy against AR.

## Materials and methods

### Ethical approval

All experiments using mice were approved by the Institutional Review Committee of Juntendo University (approval no 2020136, 2021196, 2022107) and carried out in accordance with relevant guidelines and regulations. All methods are reported in accordance with ARRIVE guidelines.

### Mice

*CD300f*^*flox/flox*^ mice with a C57BL/6J background were generated, in which the targeted gene region is sandwiched by loxP sequences recognized by Cre recombinase (TransGenic Inc., Kobe, Japan). Male WT, *CD300f*^*−/−*^, and *Kit*^*W−sh/W−sh*^ mice with a BALB/c background (aged 8–10 weeks) or male *CD300f*^*flox/flox*^ and *mast cell protease 5* (*MCPT5)-Cre*^+*/−*^*CD300f*^*flox/flox*^ mice on a C57BL/6J background (aged 8–10 weeks) were used in murine models of AR^9,32^.

### Reagents

The following antibodies (Abs) were used: biotinylated anti-LMIR3/CD300f (3-14-11; rat IgG2a) (ACTGen, Nagano, Japan), fluorescein isothiocyanate (FITC)-conjugated anti-mouse CD3ε, anti-mouse/human B220, and anti-mouse Ly-6G, anti-mouse CD4, anti-mouse CD8a, anti-mouse CD11c, anti-mouse CD19, peridinin chlorophyll protein (PerCP)-conjugated anti-mouse/human CD11b, Brilliant Violet 421-conjugated anti-mouse CD11c, phycoerythrin (PE)/cyanine 7 (Cy7)-conjugated anti-mouse FcεRI (MAR-1), PerCP-Cy5.5-conjugated anti-mouse CD49b, allophycocyanin (APC)-conjugated anti-mouse c-Kit and mouse Ly-6G, APC/Cy7-conjugated anti-mouse CD45, and Brilliant Violet 450-conjugated anti-mouse CD45 (BioLegend, San Diego, CA), and anti-ceramide (MID 15B4; Enzo Life Sciences, Plymouth Meeting, PA). Mouse IgM (MOPC-104E; BioLegend, San Diego, CA) was used as a control for anti-ceramide Ab. PE-conjugated streptavidin was purchased from BioLegend. RW pollen and RW extract were purchased from PolyScience (Niles, IL) and LSL Co Ltd (Tokyo, Japan), respectively. Cytokines were obtained from R&D Systems (Minneapolis, MN). C-24 ceramide [ceramide (d18:1/24:0); C42H83NO3] was obtained from Toronto Research Chemicals Inc. (Toronto, Canada). Phosphatidylserine (PS) [1,2-Dipalmitoyl-sn-glycero-3-phosphoserine] was obtained from Echelon Biosciences Inc. (Salt Lake City, UT).

### Preparation of liposomes

Liposomes were prepared by using ultrasonic supercritical fluid system^33,34^. The system consists of a high-pressure pump for CO2 (PU–2086, Jasco, Japan), an ultrasonic device (Ultrasonic Multi Cleaner W–118, Honda Electronics Co., Japan), thermostatted water bath with sonicator at the bottom, chiller (TBG020AA, Advantec, Japan), a pressure-resistant steel reactor vessel (SUS-316; inner diameter 20.0 mm; outer diameter 25.0 mm; length 250 mm, GL Sciences, Japan), and a back-pressure regulator (BPR; AKICO, Japan). A pressure gauge (GLT–21–25 MPa, Migishita Seiki MFG. Co. Ltd., Japan) was connected between the reactor vessel and BPR to measure the pressure.

The liposomes were prepared as follows. The reactor vessel charged with distilled water and sample material was connected to the system with 1/16 inch tubing and placed horizontally in the water bath controlled at 50 °C. Carbon dioxide from a siphon-type cylinder was pumped through the chiller tubing to maintain the contents in the liquid phase. The contents were then heated in the water bath to the supercritical state before introducion into the reactor vessel. The pressure was controlled by the BPR at 20 MPa. When the temperature and pressure in the reactor vessel attained the desired condition, ultrasonication was started at 45 kHz and 600 W. After a certain time of treatment, the reactor vessel was vertically oriented for 15 min and then depressurized from the top by adjusting the BPR. The prepared liposomes were collected by opening the vessel and then kept in the refrigerator. As material for liposomes, ceramide or PS was used as material for liposomes at a concentration of 0.01 g/L. Once prepared, liposomes remain stable for at least one month.

### RW pollen-induced murine models of AR

The murine models of AR were used with some modifications^3,4,30,31,35^. Mice were sensitized twice within a 2-week interval by intradermal injection with 100 μg RW pollen plus 1 mg alum on day 0 and intraperitoneal injection with 100 μg RW pollen on day 14, and then intranasal injection with 1 mg RW pollen or phosphate buffer saline (PBS) for four consecutive days (on days 28–31). Alternatively, mice were given intradermal injection with PBS plus 1 mg alum on day 0 and intraperitoneal injection with PBS on day 14, and then intranasal injection with PBS for four consecutive days (on days 28–31) as controls. The frequency of freezing was counted for 10 min immediately after nasal challenge with RW pollen. Sampling of tissues or blood was performed one day after the final challenge with RW pollen.

### Treatment with liposomes in mouse models of AR

For intravenous treatment with antibody or liposomes in AR models, mice were intravenously injected with 5 μg of anti-ceramide Ab or control Ab or 2 μg of ceramide liposomes or distilled water as a control on day 27 and days 28, 29, 30, and 31 at 3 h before each intranasal challenge with RW pollen. For intranasal treatment with liposomes, mice were intranasally injected with 2 μg of ceramide liposomes, PS liposomes, or distilled water as a control or 5 mg of fluticasone propionate (GlaxoSmithKline, Tokyo, Japan) or 0.6% dimethyl sulfoxide (DMSO) as a control on day 27 and days 28, 29, 30, and 31 at 5 h before each intranasal challenge with RW pollen^36^. In Fig. 5f, mice were intranasally injected with 0.2, 0.6, or 2 μg of ceramide liposomes or distilled water as a control. In Fig. S6b, mice were intranasally injected with 0.4 or 2 μg of ceramide liposomes or distilled water as a control.

### Measurements for cytokines, IgE, and ragweed-specific IgE using enzyme-linked immunosorbent assay (ELISA)

ELISA kits for Interleukin-4 (IL-4), IL-5, and IL-13 (R&D Systems) were used. Ragweed-specific IgE was determined by luminescence ELISA as previously described^9,21,37^. Briefly, RW pollen extract-coated ELISA plates were blocked before adding serial dilutions of serum samples. After washing the wells, biotinylated anti-IgE Ab (R35-118) (BD Pharmingen) was added. After incubation and washing, streptavidin-HRP was added. After incubation and washing, 3,3',5,5'-Tetramethylbenzidine (TMB) substrate solution and stop solution were added (BD Biosciences, San Jose, CA). The absorbance was measured at 450 nm using a microplate reader.

### Histological analyses

Histological analyses were performed as previously described^3,4,30,31,38^. Briefly, the removed noses were fixed in 4% paraformaldehyde for 3 days and decalcified in 0.12 mol/L ethylenediaminetetraacetic acid (EDTA) solution (pH 6.5) for 2 weeks. After decalcification, paraffin-embedded sections were stained with Giemsa, Congo red, chloroacetate esterase, or toluidine blue. The numbers of mast cells and degranulated mast cells or the numbers of eosinophils were counted in the nasal mucosa lining the nasal cavity in chloroacetate esterase-stained sections or Congo red-stained sections, respectively. Both toluidine blue- and chloroacetate esterase-stained mast cells were counted as connective tissue mast cells^37^.

### Induction of passive cutaneous anaphylaxis (PCA)

PCA was performed as previously described^9,16^. Briefly, mice were injected intradermally with 50 ng anti-dinitrophenyl (DNP) IgE (H1-ε-26) into each ear. Twenty-four hours later, the mice administered an intravenous injection of Evans blue dye (Sigma-Aldrich, St Louis, MO) containing 250 μg DNP-human serum albumin. The amounts of extravasated dye at 30 min after antigen challenges were evaluated by measuring the absorbance at 620 nm wave length.

### In vitro analysis of mast cell degranulation

To generate bone marrow-derived mast cells (BMMCs), BM cells were cultured in the presence of 10 ng/mL IL-3 for 5 weeks. To analyze mast cell degranulation, the β-hexosaminidase assay^9^ was performed for BMMCs that had been sensitized with 0.5 mg/mL anti-trinitrophenyl (TNP) IgE for 12 h and then stimulated with 10 ng/mL TNP-bovine serum albumin (BSA) in the presence of 10 mg/mL ceramide liposomes, PS liposomes, or vehicle for 1 h.

### Flow cytometry

Single cell suspensions of peritoneal cells, BM cells, peripheral blood (PB) cells, spleen cells, or nasal tissue cells were analyzed by flow cytometry using FACSVerse (BD Biosciences) equipped with FlowJo software (Tree Star)^9,16,21,37^. To prepare nasal tissue cells^28^, noses were minced and digested 2 mg/mL collagenase type I (FUJIFILM) and 0.1 mg/mL DNase I (Roche) for 1 h at 37 °C. After filtrating and washing, cell suspensions were used.

### Statistical analyses

**Figure 1:**
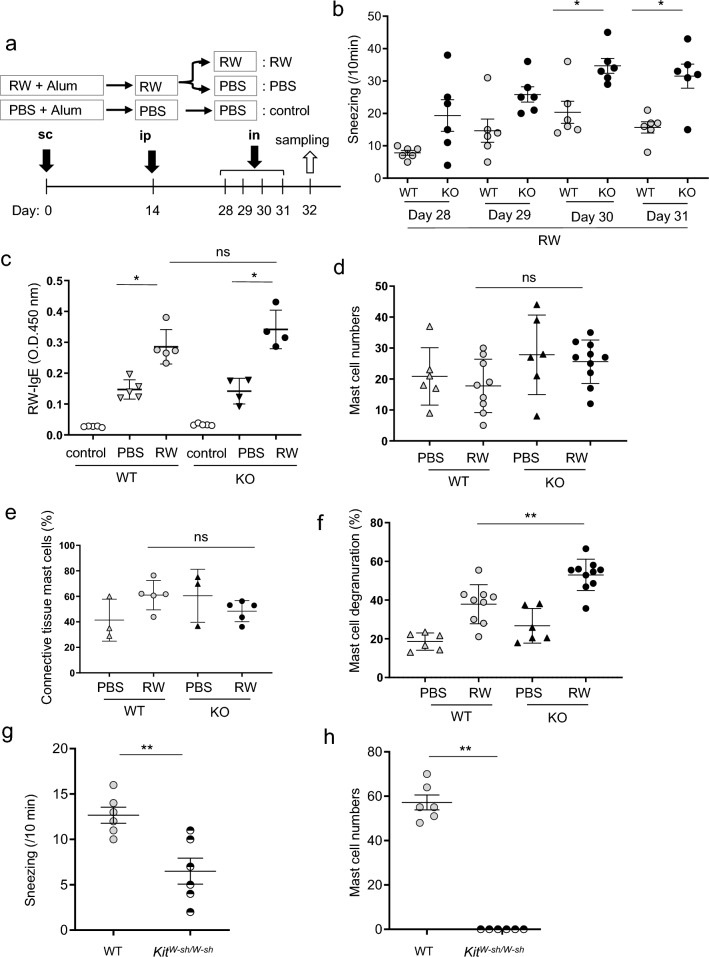
CD300f deficiency exacerbated RW pollen-induced AR in murine models. (**a**) A schematic representation of murine models of RW-induced AR. “RW” or “PBS” indicates mice given the intranasal injection of RW or PBS, respectively, after sensitization with RW. “Control” indicates mice given the intranasal injection of PBS after subcutaneous injection of alum alone and intraperitoneal injection of PBS. (**b**) The frequency of sneezing in WT and *CD300f*^*−/−*^ (KO) mice after each challenge with RW pollen on days 28, 29, 30, and 31. n = 6 per group; ± SD. Data are pooled from two independent experiments. (**c**–**g**) The levels of RW-specific IgE in serum (**c**), the numbers of mast cells (**d**), the percentages of connective tissue mast cells (**e**), and the percentages of degranulated mast cells (**f**). (**c,e**) n = 3–5 per group; ± SD. Data are representative of two independent experiments. (**d,f**) n = 6–10 per group; ± SD. Data are pooled from two independent experiments. (**g**) The frequency of sneezing in WT and *Kit*^*W−sh/W−sh*^ mice after the last challenge with RW pollen on day 32. (**h**) The numbers of mast cells in the nasal tissues from WT and *Kit*^*W−sh/W−sh*^ mice after the last challenge with RW pollen on day 32. n = 6 per group; ± SD. Data are pooled from two independent experiments. *P < 0.05 or **P < 0.01. *ns* not significant.

**Figure 2:**
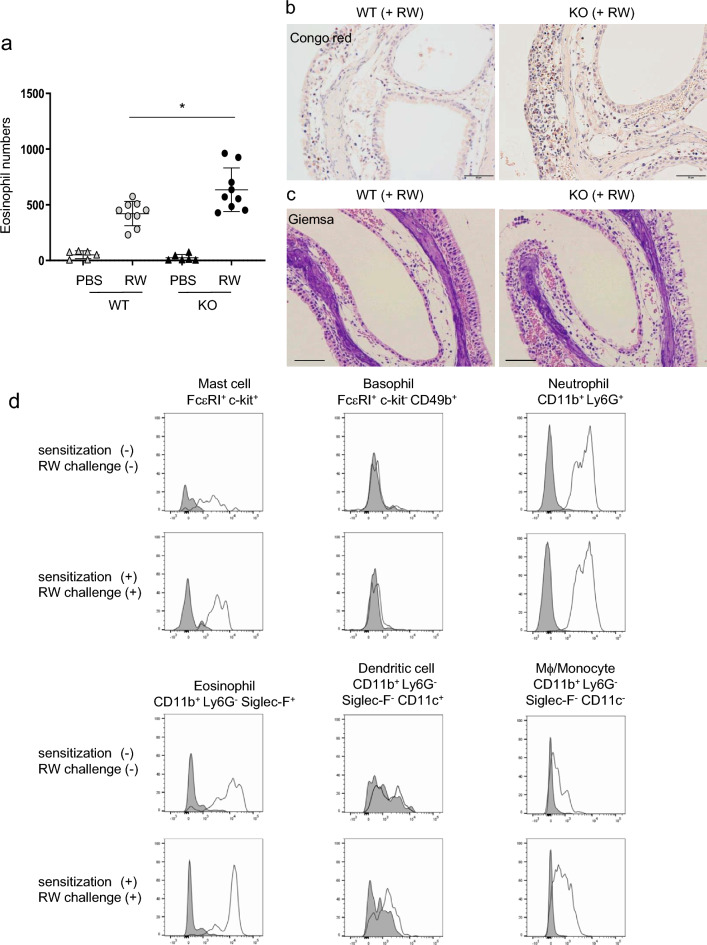
CD300f deficiency enhanced eosinophil infiltration in the nasal tissues in murine models of AR. (**a**) The numbers of eosinophils in the nasal tissues from WT and *CD300f*^*−/−*^ mice after the last challenge with RW pollen or PBS on day 32. n = 6–9 per group; ± SD. Data are pooled from two independent experiments. *P < 0.05. (**b,c**) Congo red staining (**b**) and Giemsa staining (**c**) of the nasal sections of WT mice and *CD300f*^*−/−*^ mice after the last challenge with RW pollen on day 32. (**b,c**) Scale bars, 50 μm. Data are representative of three independent experiments. (**d**) Surface expression levels of CD300f in FcεRI^+^c-Kit^+^ mast cells, FcεRI^+^c-Kit^*−*^CD49b^+^ basophils, CD11b^+^Ly-6G^+^ neutrophils, CD11b^+^Ly-6G^*−*^Siglec-F^+^ eosinophils, CD11b^+^Ly-6G^*−*^Siglec-F^*−*^CD11c^+^ dendritic cells, and CD11b^+^Ly-6G^*−*^Siglec-F^*−*^CD11c^*−*^ Mf/monocytes in the nasal tissues of WT mice under steady conditions or on day 31 in AR models. White and gray histograms indicate staining with anti-CD300f Ab and control Ab, respectively. Data are representative of three independent experiments.

**Figure 3:**
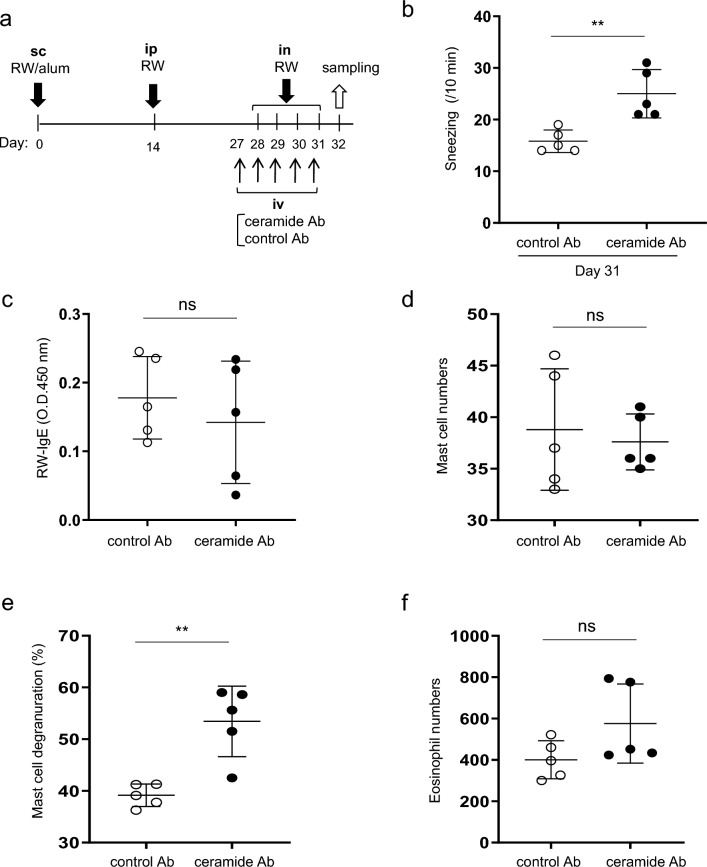
Intravenous administration of ceramide antibody exacerbated the sneezing symptom of AR in murine models. (**a**) A schematic representation of intravenous treatments with ceramide antibody or control antibody in AR models. (**b**) The frequency of sneezing in WT mice treated with ceramide antibody or control antibody after the last challenge with RW pollen on day 31. (**c**–**g**) The levels of RW-specific IgE in serum (**c**) and the numbers of mast cells (**d**) and eosinophils (**f**) and the percentages of degranulated mast cells (**e**) in the nasal tissues from ceramide antibody- or control antibody-treated WT mice after the last challenge with RW pollen on day 32. Data are representative of two independent experiments. n = 5 per group; ± SD. **P < 0.01. *ns* not significant.

**Figure 4:**
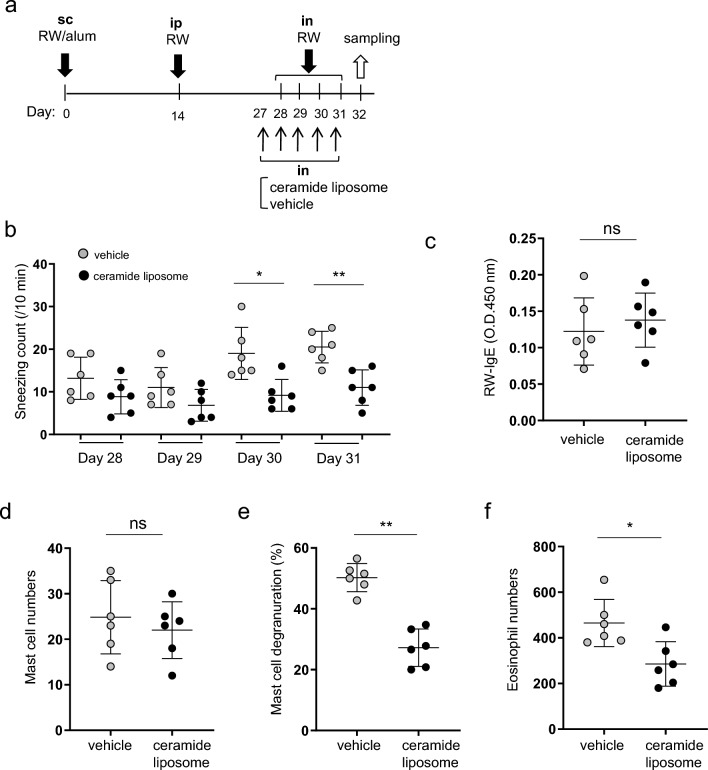
Intranasal administration of ceramide liposomes reduced the symptoms of AR in murine models. (**a**) A schematic representation of intranasal treatment with ceramide liposomes or vehicle in AR models. (**b**) The frequency of sneezing in WT mice treated with ceramide liposomes or vehicle after each challenge with RW pollen on days 28, 29, 30, and 31. (**c**–**g**) The levels of RW-specific IgE in serum (**c**) and the numbers of mast cells (**d**) and eosinophils (**f**) and the percentages of degranulated mast cells (**e**) in the nasal tissues from ceramide liposome- or vehicle-treated WT mice after the last challenge with RW pollen on day 32. Data are representative of two independent experiments. n = 6 per group; ± SD. *P < 0.05 or **P < 0.01. *ns* not significant.

**Figure 5:**
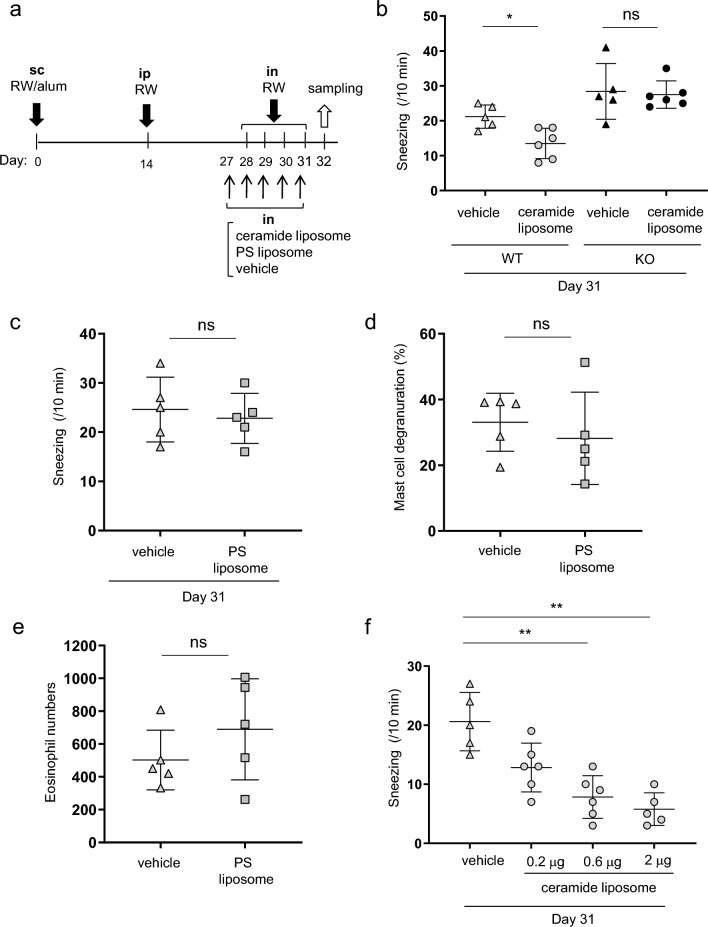
Intranasal administration of ceramide liposomes, but not of PS liposomes, reduced the frequency of sneezing by targeting CD300f in murine models of AR. (**a**) A schematic representation of treatment with ceramide liposomes, phosphatidylserine (PS) liposomes, or vehicle in AR models. (**b**) The frequency of sneezing in the ceramide liposomes- or vehicle-treated WT and *CD300f*^*−/−*^ mice after the last challenge with RW pollen on days 31. (**c**) The frequency of sneezing in WT mice treated with PS liposomes or vehicle after the last challenge with RW pollen on days 31. (**d,e**) The percentages of degranulated mast cells (**d**) and the numbers of eosinophils (**e**) in the nasal tissues from PS liposomes or vehicle-treated WT mice after the last challenge with RW pollen on day 32. (**f**) The frequency of sneezing in WT mice treated with ceramide liposomes (0.2, 0.6, 2 mg) or vehicle after the last challenge with RW pollen on days 31. Data are representative of two independent experiments. n = 5–6 per group; ± SD. *P < 0.05 or **P < 0.01. *ns* not significant.

**Figure 6:**
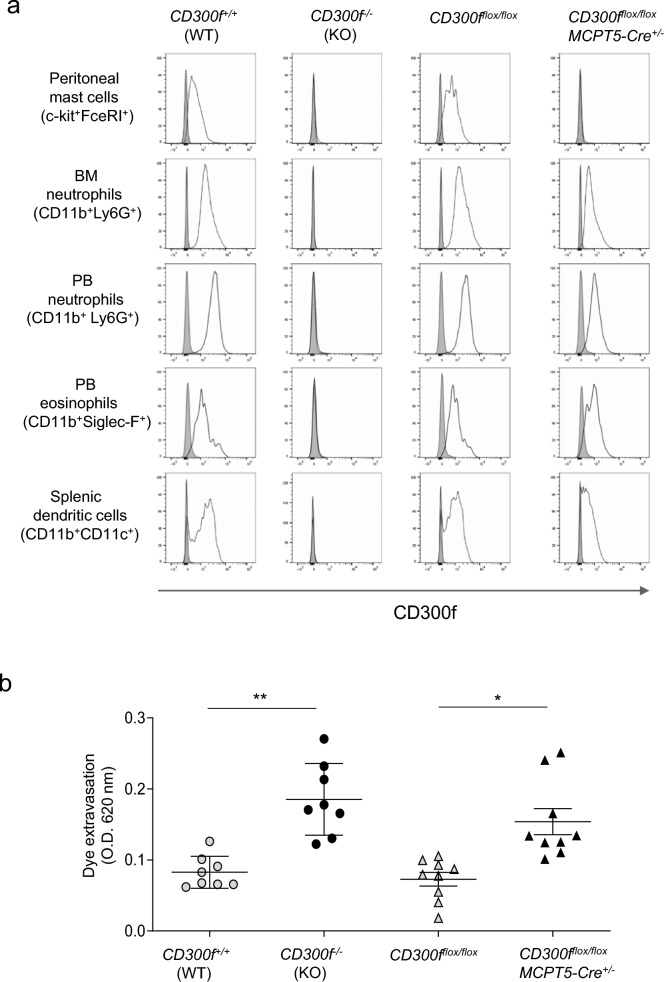
*CD300*^*flox/flox*^*MCPT5-Cre*^+*/−*^ mice exhibited loss of CD300f in MCPT5-expressing mast cells. (**a**) Surface expression levels of CD300f in FcεRI^+^c-Kit^+^ peritoneal mast cells, CD11b^+^Ly-6G^+^ BM neutrophils, CD11b^+^Ly-6G^+^ PB neutrophils, CD11b^+^Siglec-F^+^ PB eosinophils, and CD11b^+^CD11c^+^ splenic dendritic cells from WT, *CD300f*^*−/−*^ (KO), *CD300*^*flox/flox*^*,* or *CD300*^*flox/flox*^*MCPT5-Cre*^+*/−*^ mice. Data are representative of five independent experiments. (**b**) PCA responses in *CD300*^*flox/flox*^ or *CD300*^*flox/flox*^*MCPT5-Cre*^+*/−*^ mice. Data are pooled from two independent experiments. n = 8–9 per group, ± SD. *P < 0.05 or **p < 0.01.

**Figure 7:**
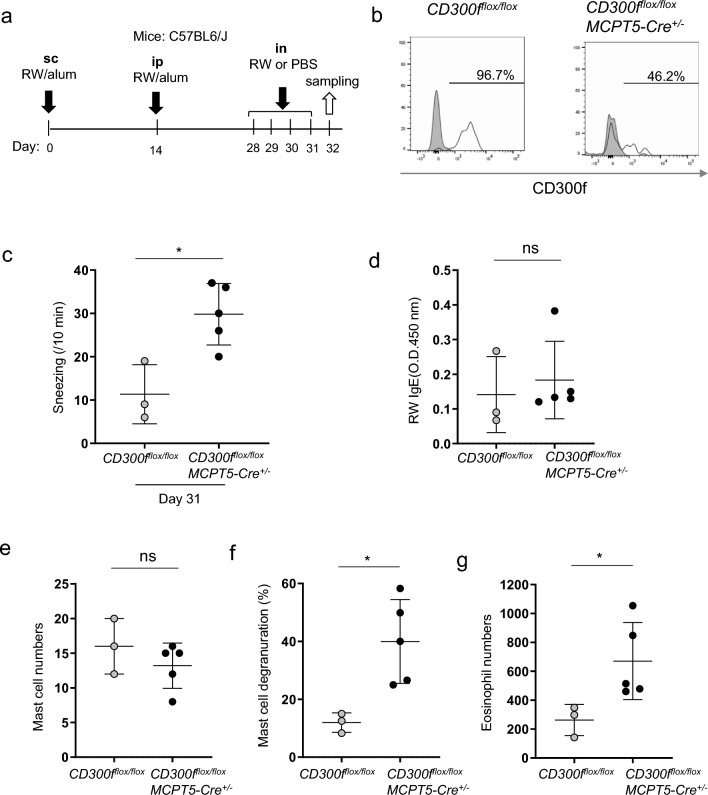
Loss of CD300f in MCPT5-expressing mast cells exacerbated the RW pollen-induced AR in murine models. (**a**) A schematic representation of RW-induced AR models in *CD300f*^*flox/flox*^*MCPT5-Cre*^+*/−*^and *CD300*^*flox/flox*^ mice in the C57BL/6J background. (**b**) The percentages of CD300f-positive nasal mast cells in *CD300f*^*flox/flox*^*MCPT5-Cre*^+*/−*^and *CD300*^*flox/flox*^ mice after the last challenge with RW pollen on day 31. (**c**) The frequency of sneezing in *CD300f*^*flox/flox*^*MCPT5-Cre*^+*/−*^ and *CD300*^*flox/flox*^ mice after the last challenge with RW pollen on day 31. (**d**–**g**) The levels of RW-specific IgE in serum (**d**) and the numbers of mast cells (**e**) and eosinophils (**g**) and the percentages of degranulated mast cells (**f**) in the nasal tissues from *CD300f*^*flox/flox*^*MCPT5-Cre*^+*/−*^ and *CD300*^*flox/flox*^ mice after the last challenge with RW pollen on day 32. Data are representative of two independent experiments. n = 3–5 per group; ± SD. *P < 0.05. *ns* not significant.

Data are expressed as mean ± standard deviation (SD). Welch’s *t*-test was used in Figs. 1g,h, 3b–f, 4c–f, 5c–e, and 7c–g, and Supplementary Fig. S2b, S3, S4b-f, and S5c-f. Brown-Forsythe and Welch analysis of variance (ANOVA) with Dunnett T3 multiple comparisons was used in Figs. 1b, 2a, 4b, 5b,f, 6b, and Fig. S5b and S6b *p < 0.05 or **p < 0.01 was considered statistically significant.

## Results

### CD300f deficiency increased the frequency of sneezing by enhancing the nasal mast cell degranulation in murine models of AR

To examine the role of an inhibitory receptor CD300f in the development of AR, we used murine models of AR. Mice were immunized twice with RW pollen on days 0 and 14 and then were intranasally challenged with RW pollen for four consecutive days on days 28 to 31 (Fig. 1a). Notably, nasal challenges with RW pollen significantly increased the frequency of sneezing during the observation period in *CD300f*^*−/−*^ mice compared with that in WT mice (Fig. 1b). Although nasal challenges with RW pollen increased RW-specific IgE levels in serum after the final challenge in both WT and *CD300f*^*−/−*^ mice compared with challenge with PBS, CD300f deficiency did not significantly affect RW-specific IgE levels in the serum after the final challenge with RW pollen (Fig. 1c). Staining of nasal tissue sections showed that the numbers of mast cells were comparable between WT and *CD300f*^*−/−*^ mice, irrespective of nasal challenges (Fig. 1d). Notably, about 40% to 60% of mast cells in the nasal tissues were connective tissue mast cells stained by both toluidine blue- and chloroacetate esterase (Fig. 1e). In contrast, the percentages of nasal mast cell degranulation were higher in *CD300f*^*−/−*^ mice than in WT mice after the last challenge with RW pollen (Fig. 1f; Supplementary Fig. S1). We confirmed that mast cell deficiency profoundly lowered the sneezing frequency in the same models (Fig. 1g,h). These results indicated that CD300f deficiency increased the frequency of sneezing presumably due to enhanced mast cell degranulation in AR models.

### CD300f deficiency increased nasal eosinophils in murine models of AR

Histological analysis showed that eosinophil numbers in the nasal tissues under steady conditions were comparable between WT and *CD300f*^*−/−*^ mice. Intranasal challenges with RW pollen increased eosinophils in the nasal tissues from both mice; however, the latter exhibited higher numbers of eosinophils and higher percentages of degranulated eosinophils in the nasal tissues than did the former (Fig. 2a–c; Supplementary Fig. S2). These results indicated that CD300f deficiency enhanced RW-induced eosinophil accumulation and degranulation in murine models of AR. Besides, flow cytometric analysis showed that CD300f was highly expressed in mast cells, eosinophils, and neutrophils in the nasal tissues under steady conditions. Intranasal challenges with RW pollen slightly increased surface expression levels of CD300f in mast cells and eosinophils (Fig. 2d). Taken together, these results indicated that CD300f deficiency accelerated the symptoms and development of AR in murine models.

### Treatment with ceramide antibody increased the frequency of sneezing in murine models of AR

To examine the role of the interaction between CD300f and its ligand ceramide in the development of AR, we intravenously injected ceramide antibody^9^ into the sensitized WT mice on days 27 to 31 before intranasal challenges in AR models (Fig. 3a). Notably, the frequency of sneezing was higher in the mice administered ceramide antibody than in those administered control antibody (Fig. 3b). Treatment with the ceramide antibody failed to influence serum levels of RW-specific IgE in WT mice (Fig. 3c). Besides, WT mice given an injection of ceramide antibody exhibited remarkable increase in degranulated mast cell number, but not in mast cell and eosinophil number, in the nasal tissues after the final challenge with RW pollen compared with those given control antibody (Fig. 3d–f). These results indicated that treatment with the ceramide antibody disrupted the in vivo CD300f-ceramide binding^9^, and increased the frequency of sneezing by enhancing mast cell degranulation.

### Intranasal administration of ceramide liposomes reduced the symptoms of AR in murine models

We next investigate if treatment with ceramide liposomes improved the development of acute AR. We confirmed that ceramide liposomes inhibited IgE-mediated degranulation of WT BMMCs, but not of CD300f-deficient BMMCs (Supplementary Fig. S3). Then, WT mice were intravenously administered ceramide liposomes on days 27–31 before intranasal challenges in the same models (Supplementary Fig. S4a). In contrast to the ceramide antibody, an intravenous administration of ceramide liposomes reduced the percentages of nasal mast cell degranulation in the nasal tissues. The same administration neither affected the serum levels of RW-specific IgE nor affected the nasal mast cell and eosinophil numbers (Fig. S4b-f).

We then asked if an intranasal administration of ceramide liposomes is effective for the improvement of AR. Importantly, this treatment on days 27–31 reduced the frequency of sneezing as well as the numbers of degranulated mast cells and eosinophils in the nasal mucosa (Fig. 4a,b,e,f). However, the intranasal administration of ceramide liposomes did not significantly alter RW-specific IgE levels in serum and nasal mast cell numbers (Fig. 4c,d). Similarly to the case of ceramide liposomes, intranasally administration of corticosteroid (fluticasone propionate) reduced the frequency of sneezing and the numbers of degranulated mast cells and eosinophils in the nasal tissues^38^; however, the same treatment neither significantly influenced serum levels of RW-specific IgE nor the nasal mast cell numbers (Supplementary Fig. S5). Thus, intranasal administration of ceramide liposomes reduced the symptoms of AR in murine models.

### Intranasal administration of ceramide liposomes, but not of PS liposomes, reduced the frequency of sneezing by targeting CD300f in murine models of AR

We found that the intranasal administration of ceramide liposomes failed to influence the frequency of sneezing in CD300f-deficient mice (Fig. 5a,b). Besides, we confirmed that the intranasal administration of phosphatidylserine neither affect the frequency of sneezing nor the numbers of degranulated mast cells and eosinophils in the nasal tissues (Fig. 5c–e). In addition, the intranasal administration of ceramide liposomes (0.2, 0.6, 2 mg) dose-dependently reduced the frequency of sneezing (Fig. 5f). Although treatment with ceramide antibody increased the frequency of sneezing in WT mice in AR models, the increased frequency was reduced by the intranasal administration of ceramide liposomes (Supplementary Fig. S6). Collectively, these results indicated that intranasal administration of ceramide liposomes increased the local levels of CD300f ligands and inhibited the IgE-mediated mast cell degranulation in the nasal tissues by targeting CD300f, thereby improving the clinical symptoms of AR.

### Loss of CD300f in MCPT5-expressing mast cells in *CD300f*^*flox/flox*^*MCPT5-Cre*^+*/−*^ mice

To clarify the role of mast cell-expressing CD300f in the development of AR, we generated conditional CD300f knock-out (*CD300f*^*flox/flox*^) mice mediated by the Cre/lox system, in which CD300f expression is lost in Cre recombinase-expressing cells under the control of a cell type-specific promoter (Supplementary Fig. S7). We crossed *CD300f*^*flox/flox*^ mice with *MCPT5-Cre*^+*/−*^ mice^32^ to generate *CD300f*^*flox/flox*^*MCPT5-Cre*^+*/−*^ mice in the genetic background of C57BL/6J, which supposedly lack CD300f expression in MCPT5-expressing mast cells. Flow cytometric analysis demonstrated that CD300f expression is lost in peritoneal FcεRIα^+^c-kit^+^ mast cells from *CD300f*^*flox/flox*^*MCPT5-Cre*^+*/−*^ mice. In contrast, we found sufficient expression of CD300f in CD11b^+^Ly6G^+^ neutrophils from BM and PB, in CD11b^+^Siglec-F^+^ eosinophils from PB, and in CD11b^+^CD11c^+^ dendritic cells from spleen in *CD300f*^*flox/flox*^*MCPT5-Cre*^+*/−*^ mice (Fig. 6a). Besides, *CD300f*^*flox/flox*^*MCPT5-Cre*^+*/−*^ mice exhibited enhanced dye extravasation in ear skin compared with *CD300f*^*flox/flox*^ mice in IgE-dependent PCA models (Fig. 6b), confirming that MCPT5-expressing mast cell-specific loss of CD300f enhanced the PCA responses^9^.

### Loss of CD300f in MCPT5-expressing mast cells in mice increased the frequency of sneezing in AR models

We then used murine models of AR in *CD300f*^*flox/flox*^*MCPT5-Cre*^+*/−*^ and *CD300f*^*flox/flox*^ mice (Fig. 7a). Consistent with our result (Fig. 1f), flow cytometric analysis showed that CD300f expression was lost in approximately 50% of nasal mast cells, which were supposed to be connective tissue mast cells, in *CD300f*^*flox/flox*^*MCPT5-Cre*^+*/−*^ mice (Fig. 7b). The frequency of sneezing after the last challenge with RW pollen was significantly higher in *CD300f*^*flox/flox*^*MCPT5-Cre*^+*/−*^ mice than in *CD300f*^*flox/flox*^ mice (Fig. 7c). Serum levels of RW-specific IgE and nasal mast cell numbers were comparable between these two types of mice (Fig. 7d,e). Importantly, MCPT5-expressing mast cell-specific loss of CD300f enhanced mast cell degranulation and increased eosinophil numbers in the nasal tissues (Fig. 7f,g). These results suggested that nasal mast cell-expressing CD300f plays critical roles in inhibiting the development of AR in murine models.

## Discussion

In the present study, we used murine models of RW pollen-induced AR^3,4,30,31,35^. The engagement of RW-specific IgE-bound FcεRI with RW pollen has been reported to trigger nasal mast cell degranulation, resulting in sneezing, a major symptom of AR, by the local release of chemical mediators in murine models^1–4,30,31^. Interestingly, we demonstrated that CD300f deficiency increased the frequency of sneezing as well as the percentages of nasal mast cell degranulation in mice, whereas it did not significantly influence the RW-specific IgE production or nasal mast cell numbers. Mast cell deficiency strongly mitigated the sneezing symptom in AR models. Besides, CD300f was highly expressed in mast cells, but not in basophils, in murine nasal tissues. Considering the inhibitory functions of CD300f in mast cells^9,16^, it is therefore plausible that CD300f suppresses the FcεRI -mediated mast cell degranulation in nasal tissues, thereby alleviating the AR symptoms in murine models. Moreover, per our previous results that CD300f-ceramide binding inhibits the IgE-driven mast cell activation^9,16,17,21,22^, intravenous administration of ceramide antibody or ceramide liposomes increased or decreased the sneezing symptom presumably by interfering with the CD300f-ceramide interaction or increasing the levels of ceramide surrounded by nasal mast cells, respectively. Intriguingly, loss of CD300f also enhanced eosinophil infiltration and degranulation in the nasal tissues in AR models: activated eosinophils are thought to induce the damage of the nasal tissues, which is associated with nasal congestion^1,2^. Importantly, CD300f deficiency only in MCPT5-expressing mast cells, corresponding to connective tissue mast cells, increased the frequency of sneezing, the percentages of nasal mast cell degranulation, and the numbers of eosinophils infiltrating into the nasal tissues in AR models. It should be noted that comparable numbers of connective tissue mast cells and mucosal mast cells distribute in the nasal tissues. In addition, it was recently reported that eotaxin (CCL11) and its receptor CCR3 are critical in nasal eosinophil accumulation in AR^39,40^. Histamine released from degranulated mast cells induces local production of eotaxin in endothelial cells^41^. Moreover, histamine, adenosine, or tryptase released from degranulated mast cells directly activates eosinophils^42^. Accordingly, it is reasonable to conclude that the mast cell-expressing CD300f inhibited the FcεRI -mediated mast cell degranulation in the nasal tissues, thereby suppressing local infiltration of eosinophils in AR models. Given that CD300f inhibits eotaxin-induced eosinophil activation^20^, it seems possible that eosinophil-expressing CD300f also suppressed eosinophil accumulation to a certain degree in AR models.

Most importantly, we demonstrated that the intranasal administration of ceramide liposomes, but not PS liposomes, suppressed the sneezing symptoms as well as mast cell degranulation and eosinophil infiltration in the nasal tissues in AR models. Taken together, the results suggest that ceramide liposomes inhibit nasal mast cell degranulation predominantly by targeting the nasal mast cell-expressing CD300f, which in turn suppresses eosinophil infiltration. Because CD300f was highly expressed in nasal eosinophils, the interaction between eosinophil-expressing CD300f and ceramide may also play a certain role in inhibiting eosinophil accumulation. Consistent with enhanced expression of CD300f in PB eosinophils from AR patients^20^, we found increased expression of CD300f in nasal mast cells and eosinophils from mice with AR, implying that CD300f down-regulates the development of AR. In any case, intranasal administration of ceramide liposomes suppressed the development of AR in murine models by targeting CD300f. However, we should consider that cellular and extracellular ceramides have a variety of biological functions in various tissues that are associated with the length and saturation of fatty acyl chains in the ceramides^43,44^. Endogenous intracellular ceramides appear to be different from exogenous ceramides, such as ceramide (d18:1/24:0) liposomes used in this study. In contrast, endogenous extracelluar ceramides such as those present in lipoproteins may act as CD300f ligands, similarly to ceramide liposomes. Cell-permeable short-chain ceramides have various functions^43–46^. For example, C6-ceramide suppresses ERK activation in FceRI-stimulated RBL-2H3 cells by activating a proteins tyrosine phosphatase^45^. Additionally, C2-ceramide inhibits the degranulation in FceRI-stimulated RBL-2H3 cells by suppressing phospholipase D activation^46^. We cannot rule out the possibility that these actions are also involved in the inhibition of mast cell activation by ceramide (d18:1/24:0) liposomes; however, cell-permeable short-chain ceramides have biological properties different from those of natural species^43,44^.

In the present study, we prepared ceramide liposomes by using ultrasonic supercritical fluid system^33,34^_._ Accordingly, the improvement of this method will be critical for the clinical application of ceramide liposomes as a therapeutic strategy against AR. This treatment is expected to have an immediate effect on the AR symptoms. Notably, the intranasal administration of corticosteroid or ceramide liposomes showed similar treatment effects on AR in murine models. Although considerable controversy exists over whether glucocorticoids inhibit histamine release from mast cells^47^, it was reported that glucocorticoids rapidly inhibit IgE-mediated exocytosis of mast cells by reducing [Ca^2+^]_i_ elevation^48^. Because these two treatments are effective against AR through different molecular mechanisms, the combination therapy with ceramide liposomes and corticosteroid may be a promising treatment against AR^1,2^. Nonetheless, we should measure comprehensively ceramide levels in the nasal tissues before and after the intranasal administration of ceramide liposomes in the next step of our research, thereby clarifying how ceramide liposomes are absorbed into and retained on the nasal tissues.

It should be noted that in the present study, CD300f seemed to play a negligible role in antigen-specific IgE production in our models of AR. The route of RW administration and/or the presence of alum adjuvant in RW administration may affect Th2/ T follicular helper (Tfh) responses via CD300f. Analysis of mice deficient in CD300f specifically in immune cells (e.g., dendritic cells) will be required for further investigation.

In conclusion, CD300f inhibits the symptoms and progression of AR in murine models. Intranasal administration of ceramide liposomes might be an attractive therapeutic strategy against AR.

### Supplementary Information

Supplementary Figures.

## Data Availability

The datasets generated and/or analyzed during the current study are available from the corresponding authors on reasonable request.
