# Supplementary material for: Intranasal administration of ceramide liposome suppresses allergic rhinitis by targeting CD300f in murine models
Source: Sci Rep. 2024 Apr 10;14:8398. doi: 10.1038/s41598-024-58923-w (PMC11006841; doi:10.1038/s41598-024-58923-w)
Supplement: Supplementary file 1 — Supplementary Figures. [file 41598_2024_58923_MOESM1_ESM.pdf]

Supplemental Material

Intranasal administration of ceramide liposome suppresses allergic rhinitis by targeting CD300f in murine models

Takuma Ide[1,2], Kumi Izawa[#1], Wahyu Diono[3], Anna Kamei[1,4], Tomoaki Ando[1], Ayako Kaitani[1], Akie Maehara[1], Akihisa Yoshikawa[1,2], Risa Yamamoto[1], Shino Uchida[1,5], Hexing Wang[1,4], Mayuki Kojima[1,6], Keiko Maeda[1,7], Nobuhiro Nakano[1], Nakamura Masahiro[2], Toshiaki Shimizu[1,6], Hideoki Ogawa[1], Ko Okumura[1], Fumihiko Matsumoto[2], Katsuhisa Ikeda[2], Motonobu Goto[3], Jiro Kitaura[#1,4]

[1]Atopy (Allergy) Research Center, Juntendo University Graduate School of Medicine, 2-1-1 Hongo, Bunkyo-ku, Tokyo 113-8421, Japan

[2]Department of Otorhinolaryngology, Juntendo University Graduate School of Medicine, 2-1-1 Hongo, Bunkyo-ku, Tokyo 113-8421, Japan

[3]Department of Materials Process Engineering, Nagoya University, Furo-cho, Chikusa-ku, Nagoya 464-8603, Japan

[4]Department of Science of Allergy and Inflammation, Juntendo University Graduate School of Medicine, 2-1-1 Hongo, Bunkyo-ku, Tokyo 113-8421, Japan

[5]Department of Gastroenterology Immunology, Juntendo University Graduate School of Medicine, 2-1-1 Hongo, Bunkyo-ku, Tokyo 113-8421, Japan

[6]Department of Pediatrics and Adolescent Medicine, Juntendo University Graduate School of Medicine, 2-1-1 Hongo, Bunkyo-ku, Tokyo 113-8421, Japan

[7]Department of Immunological Diagnosis, Juntendo University Graduate School of Medicine, 2-1-1 Hongo, Bunkyo-ku, Tokyo 113-8421, Japan.

#Corresponding authors:

Kumi Izawa, MD, PhD, Atopy (Allergy) Research Center, Juntendo University Graduate School of Medicine, 2-1-1 Hongo, Bunkyo-ku, Tokyo 113-8421, Japan; Phone: (+81-3) 5802-1591; Fax: (+81-3) 3813-5512; email: k-izawa@juntendo.ac.jp

Jiro Kitaura, MD, PhD, Atopy (Allergy) Research Center, Juntendo University Graduate School of Medicine, 2-1-1 Hongo, Bunkyo-ku, Tokyo 113-8421, Japan; Phone: (+81-3) 5802-1591; Fax: (+81-3) 3813-5512; email: j-kitaura@juntendo.ac.jp

Figure S1. CD300f deficiency enhanced mast cell degranulation in AR models. The degranulated mast cells were counted in the nasal mucosa lining the nasal cavity in chloroacetate esterase-stained sections. Representative imaging of non-degranulated or degranulated mast cells from WT or *CD300f*$^{-/-}$ mice after the last challenge with RW pollen on day 32 in acute AR models. Scale bars, 20 μm.

Figure S2. CD300f deficiency enhanced eosinophil degranulation in AR models. (**a**) The percentages of degranulated eosinophils in the nasal tissues from WT and *CD300f*$^{-/-}$ (KO) mice after the last challenge with RW pollen on day 32. n = 4-5 per group; ± SD. Data are representative of two independent experiments. *$P < 0.05$. (**b**) The degranulated eosinophils were counted in the nasal mucosa lining the nasal cavity in Congo red-stained sections. Representative imaging of non-degranulated or degranulated eosinophils. Scale bars, 5 μm.

Figure S3. Ceramide liposomes inhibited IgE-mediated degranulation of BMMCs. The percentages of degranulation in anti-TNP IgE-sensitized WT and CD300f-deficient BMMCs stimulated by 10 ng/mL TNP-BSA in the presence of 10 μg/mL ceramide or phosphatidylserine (PS) liposomes or vehicle. n = 3; ± SD. Data are representative of three independent experiments. *$P < 0.05$.

Figure S4. Intravenous administration of ceramide liposomes alleviated the sneezing symptom in AR models. (**a**) A schematic representation of intravenous treatment with

ceramide liposomes or vehicle in AR models. (**b**) The frequency of sneezing in WT mice treated with ceramide liposomes or vehicle after the last challenge with RW pollen on day 31. (**c-f**) The levels of RW-specific IgE in serum (**c**) and the numbers of mast cells (**d**) and eosinophils (**f**) and the percentages of degranulated mast cells (**e**) in the nasal tissues from ceramide liposomes- or vehicle-treated WT mice after the last challenge with RW pollen on day 32. Data are representative of two independent experiments. n = 5 per group; ± SD. $*P < 0.05$. ns, not significant.

Figure S5. Intranasal administration of corticosteroid alleviated the sneezing symptom of AR in murine models. (**a**) A schematic representation of intranasal treatment with corticosteroid (fluticasone propionate) or vehicle in AR models. (**b**) The frequency of sneezing in WT mice treated with fluticasone propionate or vehicle after each challenge with RW pollen on days 28, 29, 30, and 31. (**c-f**) The levels of RW-specific IgE in serum (**c**) and the numbers of mast cells (**d**) and eosinophils (**f**) and the percentages of degranulated mast cells (**e**) in the nasal tissues from fluticasone propionate- or vehicle-treated WT mice after the last challenge with RW pollen on day 32. Data are representative of two independent experiments. n = 5-6 per group; ± SD. $*P < 0.05$ or $**P < 0.01$. ns, not significant.

Figure S6. Enhanced RW-induced sneezing in WT mice by treatment with ceramide antibody was canceled by the intranasal administration of ceramide liposomes. (**a**) A schematic representation of intravenous treatment with ceramide antibody or control

antibody and intranasal administration of ceramide liposomes or vehicle in AR models. (**b**) The frequency of sneezing in WT mice treated with ceramide antibody or control antibody and intranasally administered with ceramide liposomes or vehicle after the last challenge with RW pollen on day 31. Data are pooled from two independent experiments. n = 6 per group; ± SD. $^{**}P < 0.01$.

Figure S7. Generation of conditional CD300f knock-out ($CD300^{flox/flox}$) mice. The strategy for generating the $CD300f^{\mathrm{flox/flox}}$ mouse line is depicted. Cre recombinase under the control of a cell type-specific promoter induces the specific knock-out of CD300f in mice.

Figure S1

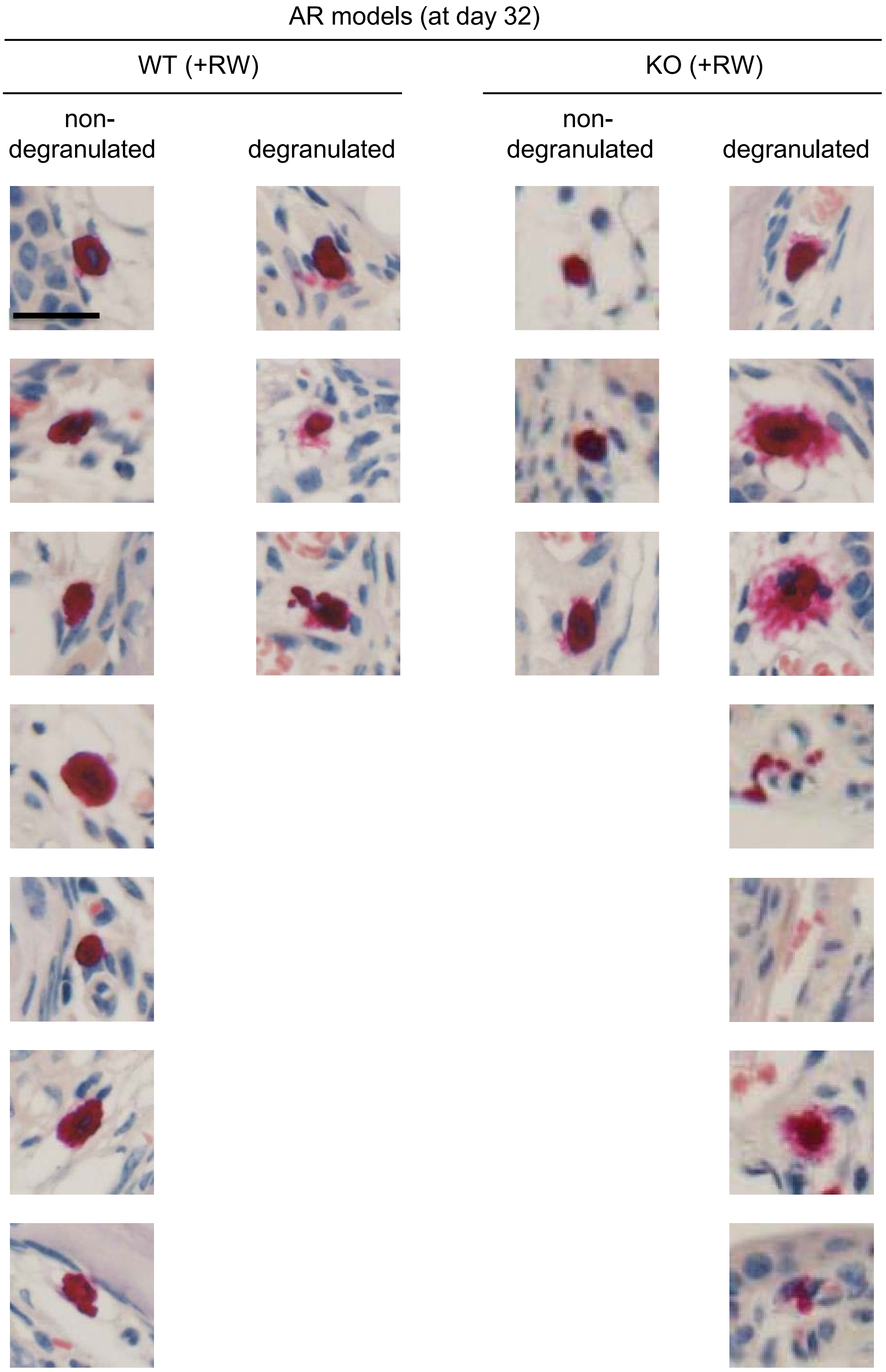
AR models (at day 32)
WT (+RW)
KO (+RW)
non-degranulated
degranulated
non-degranulated
degranulated

Figure S2

a

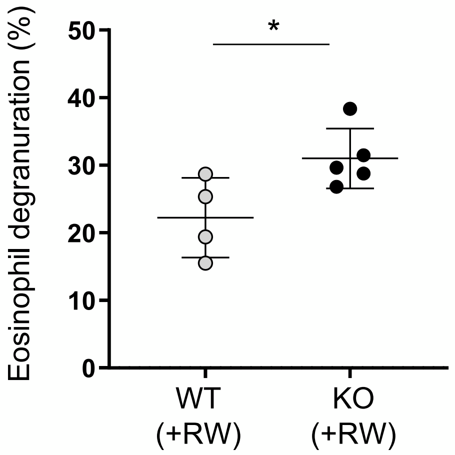
Eosinophil degranuration (%)
50
40
30
20
10
0
*
WT (+RW)
KO (+RW)

b

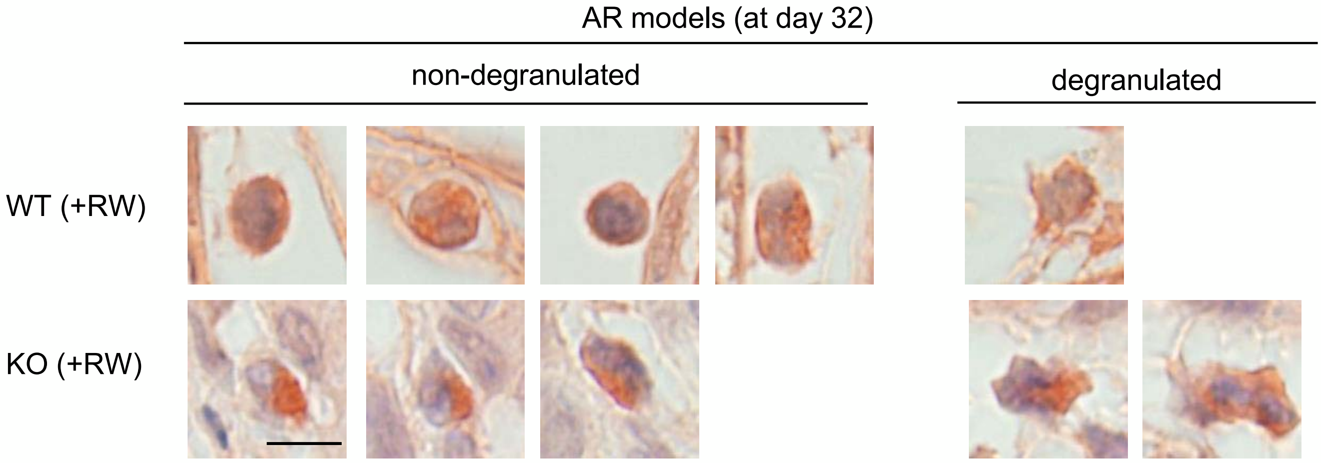
AR models (at day 32)
non-degranulated
degranulated
WT (+RW)
KO (+RW)

Figure S3

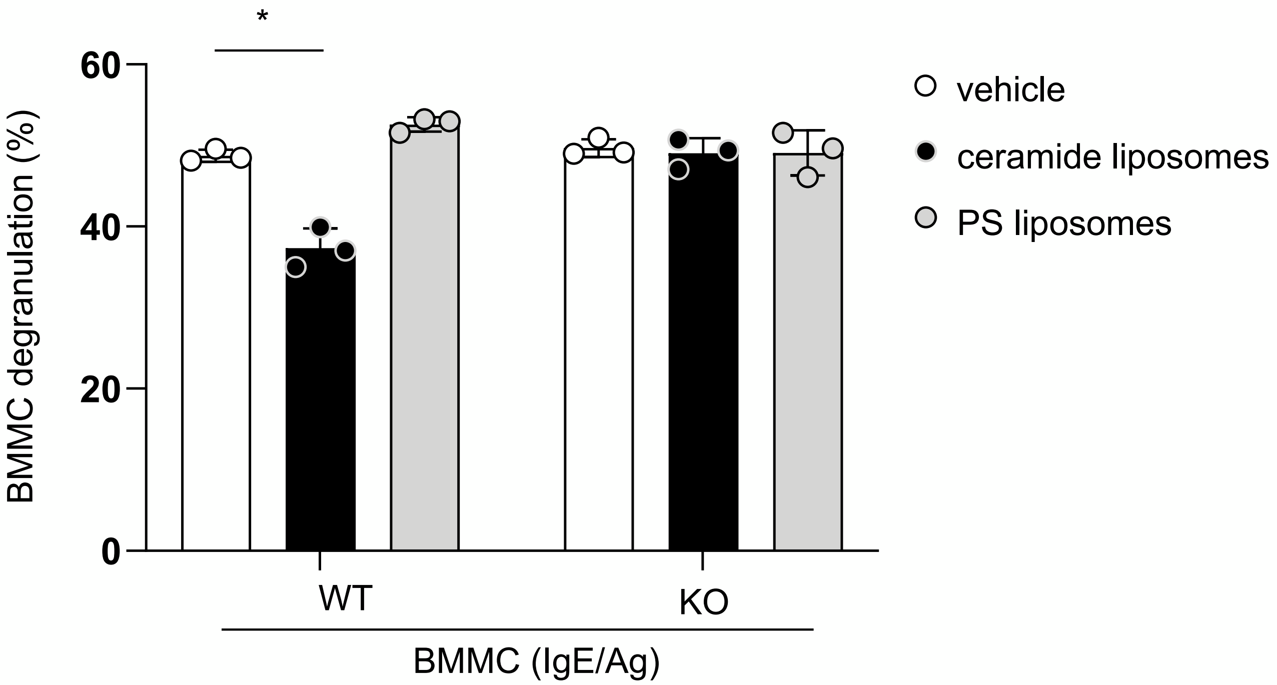
*
60
40
20
0
BMMC degranulation (%)
WT
KO
BMMC (IgE/Ag)
vehicle
ceramide liposomes
PS liposomes

Figure S4

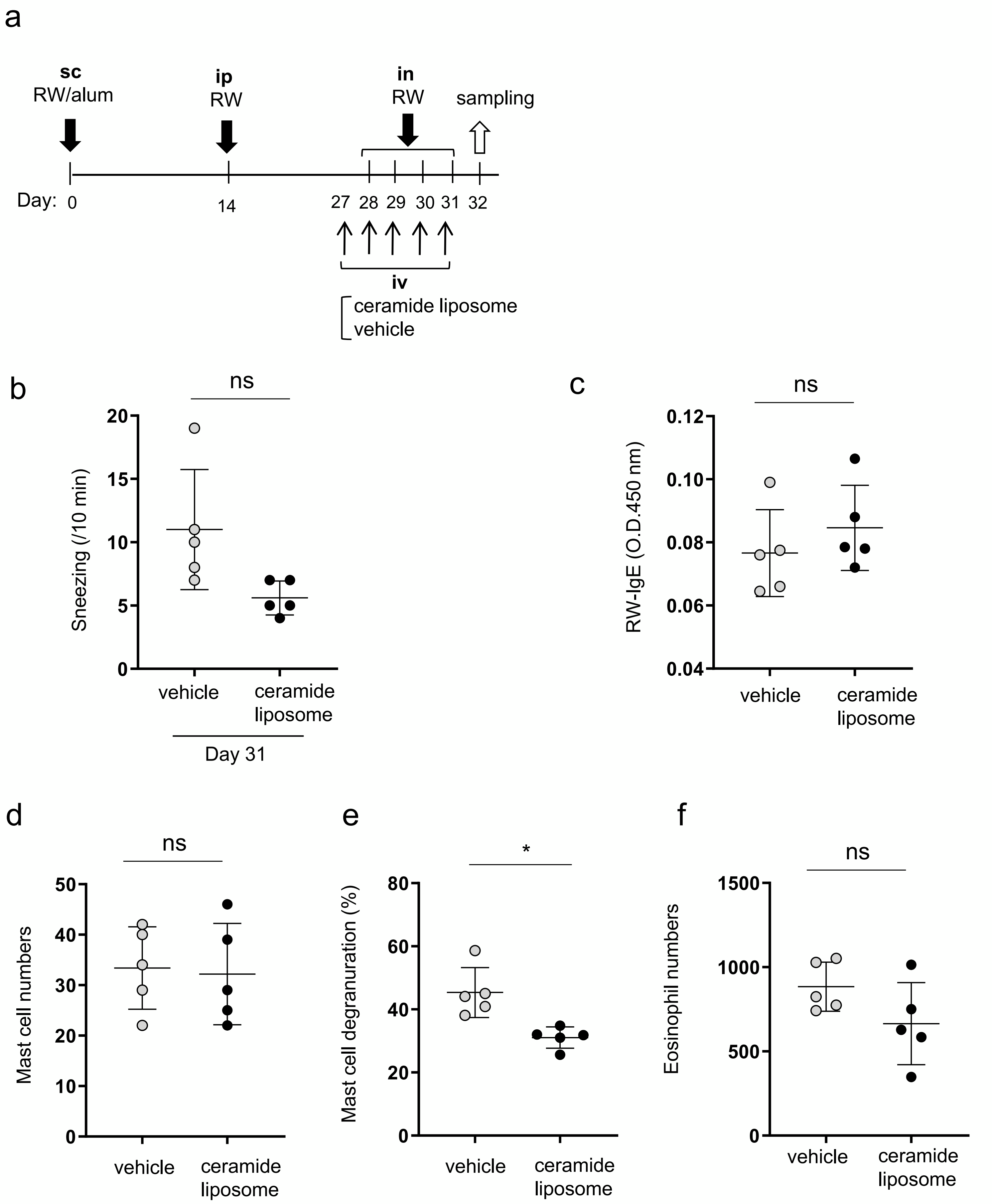

a
sc
RW/alum
ip
RW
in
RW
sampling
Day: 0
14
27 28 29 30 31 32
iv
ceramide liposome
vehicle
b
ns
Sneezing (/10 min)
20
15
10
5
0
vehicle
ceramide liposome
Day 31
c
ns
RW-IgE (O.D.450 nm)
0.12
0.10
0.08
0.06
0.04
vehicle
ceramide liposome
d
ns
Mast cell numbers
50
40
30
20
10
0
vehicle
ceramide liposome
e
*
Mast cell degranuration (%)
80
60
40
20
0
vehicle
ceramide liposome
f
ns
Eosinophil numbers
1500
1000
500
0
vehicle
ceramide liposome

Figure S5

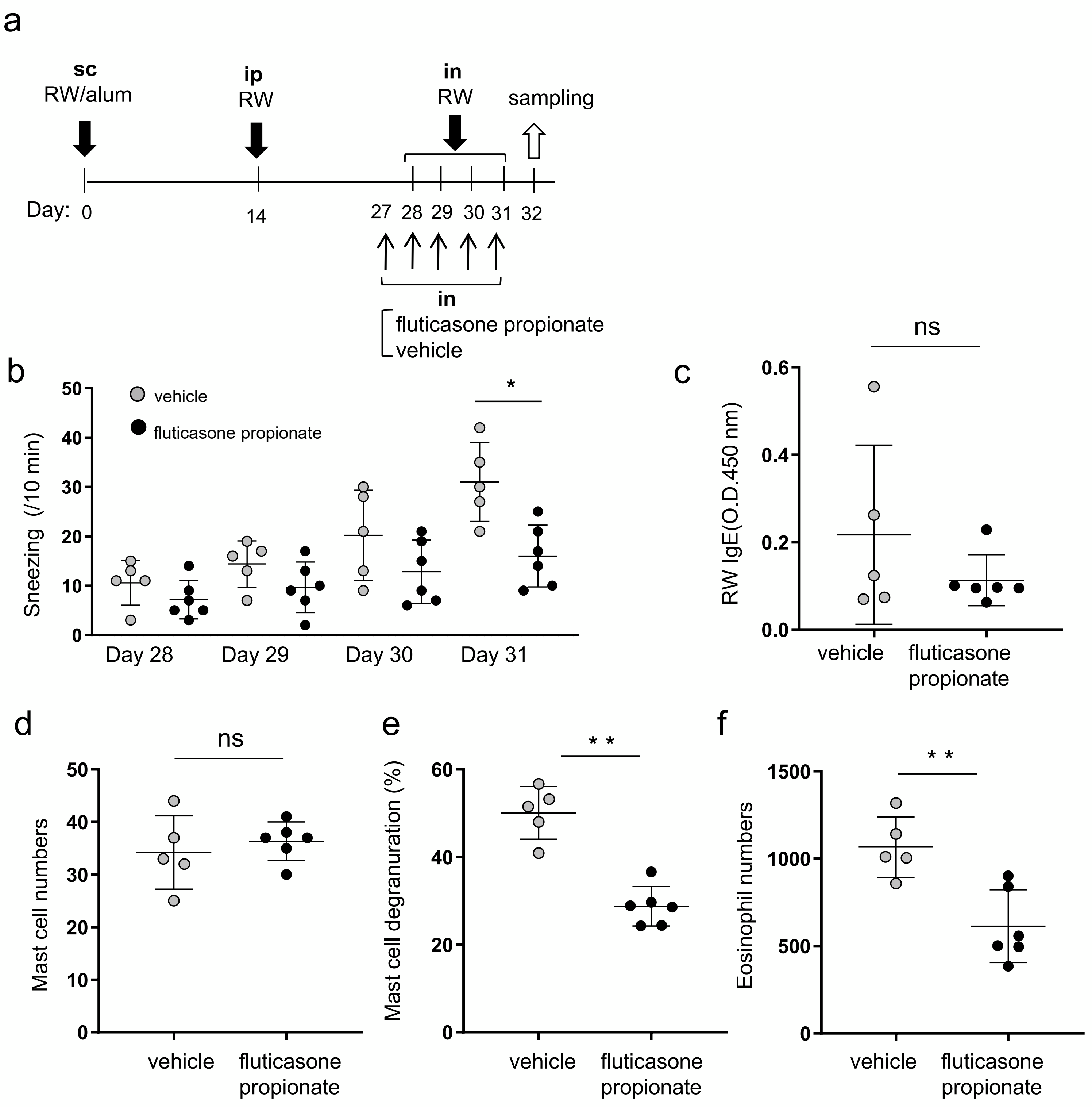
a
sc
RW/alum
ip
RW
in
RW
sampling
Day: 0
14
27 28 29 30 31 32
in
fluticasone propionate
vehicle
b
vehicle
fluticasone propionate
Sneezing (/10 min)
*
Day 28
Day 29
Day 30
Day 31
c
ns
RW IgE(O.D.450 nm)
vehicle
fluticasone propionate
d
ns
Mast cell numbers
vehicle
fluticasone propionate
e
* *
Mast cell degranuration (%)
vehicle
fluticasone propionate
f
* *
Eosinophil numbers
vehicle
fluticasone propionate

Figure S6

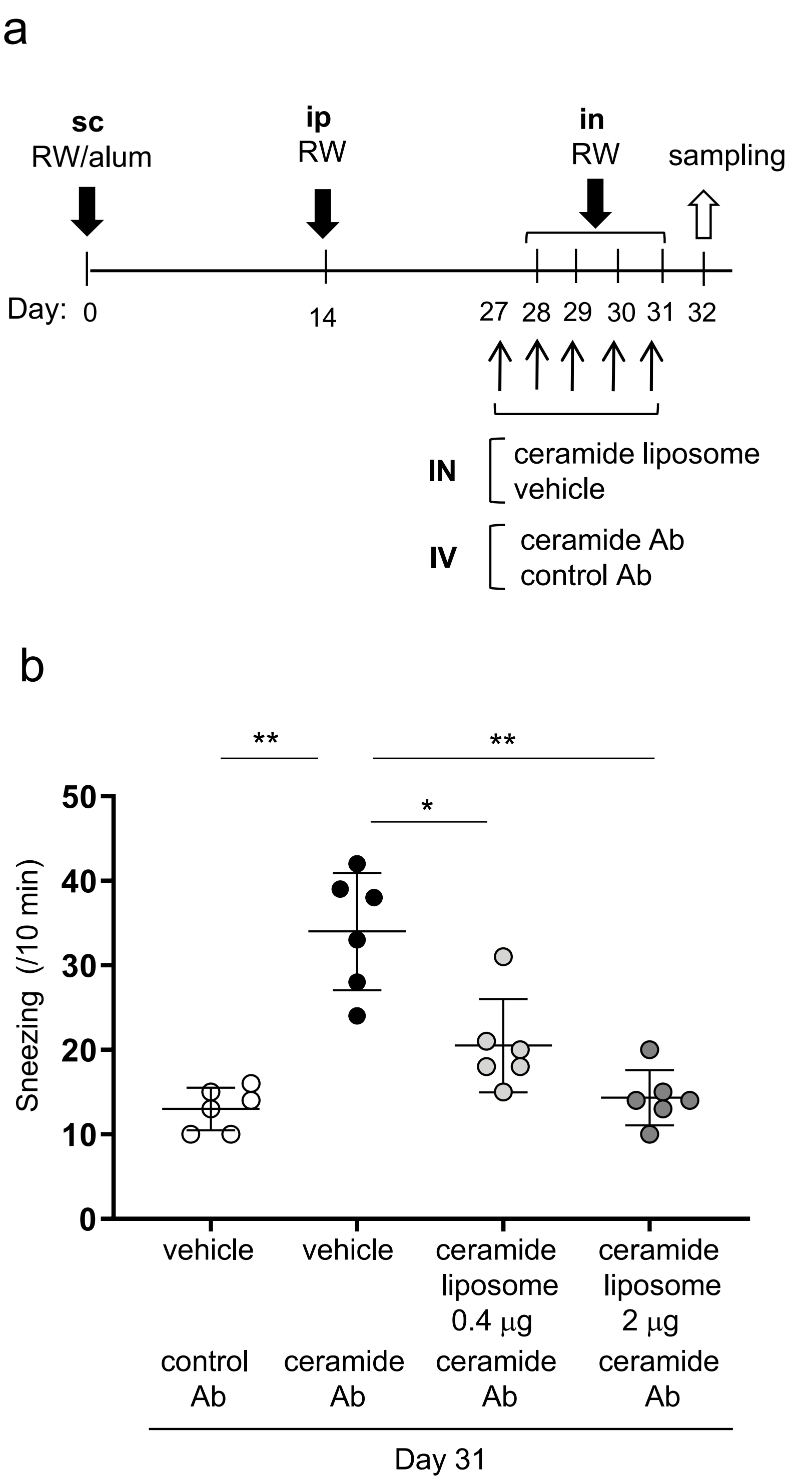
a
sc
RW/alum
ip
RW
in
RW
sampling
Day: 0
14
27 28 29 30 31 32
IN
ceramide liposome
vehicle
IV
ceramide Ab
control Ab
b
**
**
*
Sneezing (/10 min)
50
40
30
20
10
0
vehicle
vehicle
ceramide liposome 0.4 µg
ceramide liposome 2 µg
control Ab
ceramide Ab
ceramide Ab
ceramide Ab
Day 31

Figure S7

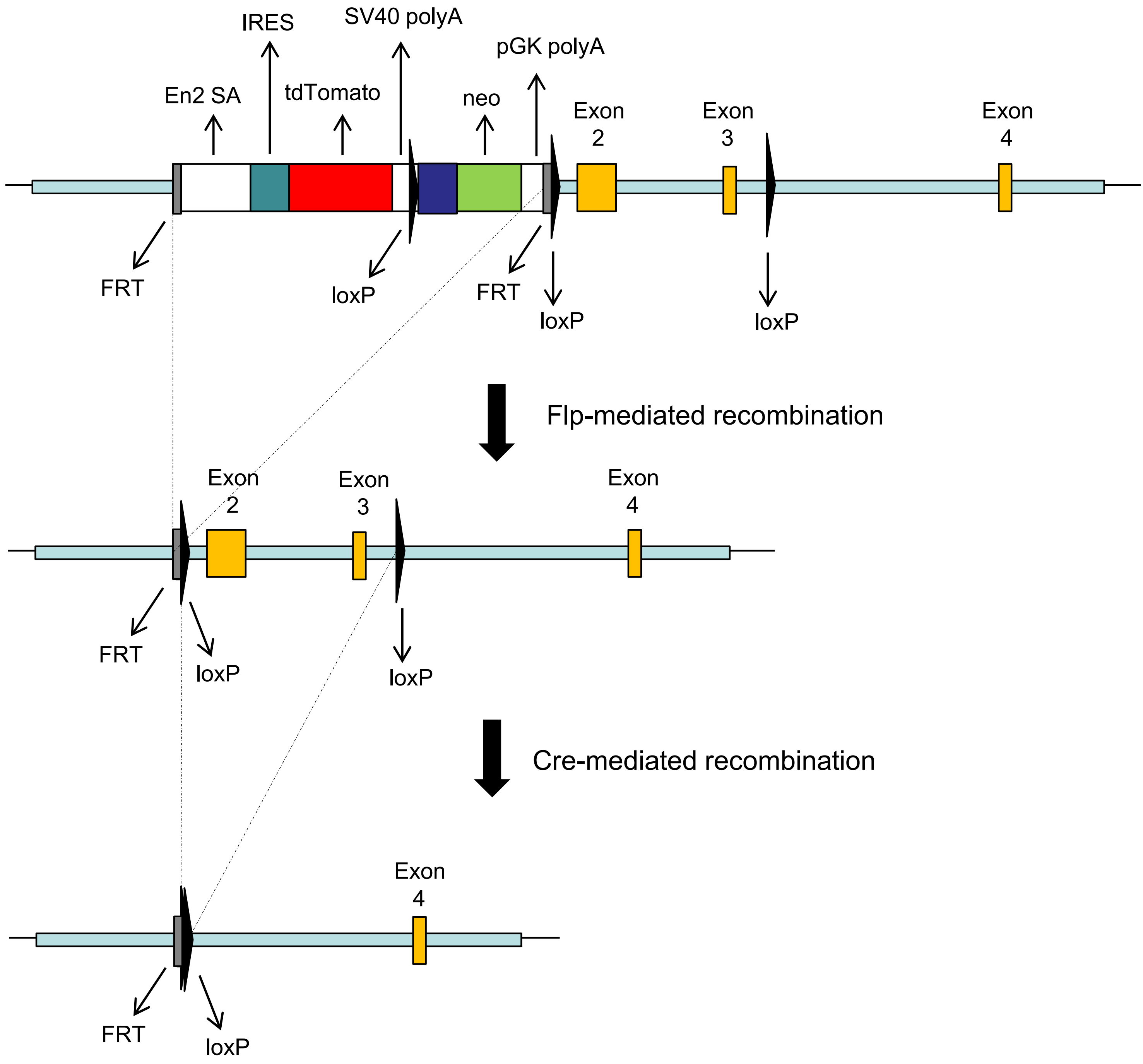
IRES
SV40 polyA
pGK polyA
En2 SA
tdTomato
neo
Exon 2
Exon 3
Exon 4
FRT
loxP
FRT
loxP
loxP
Flp-mediated recombination
Exon 2
Exon 3
Exon 4
FRT
loxP
loxP
Cre-mediated recombination
Exon 4
FRT
loxP
